# (*E*)-*N*′-(5-Bromo-2-hy­droxy­benzyl­idene)-4-(dimethyl­amino)­benzohydrazide

**DOI:** 10.1107/S1600536812013785

**Published:** 2012-04-04

**Authors:** En-Yu Wei

**Affiliations:** a Zibo Vocational Institute, Zibo 255314, People’s Republic of China

## Abstract

The title compound, C_16_H_16_BrN_3_O_2_, crystallized with two independent molcules in the asymmetric unit. Each mol­ecule has an *E* conformation about the C=N bond and the dihedral angles between the benzene rings are 30.5 (3) and 28.7 (3)°. In each mol­ecule, there is an O—H⋯N hydrogen bond and the two mol­ecules are linked by an N—H⋯O hydrogen bond. In the crystal, mol­ecules are further linked *via* N—H⋯O hydrogen bonds into chains propagating along [001].

## Related literature

For further details concerning benzohydrazone compounds, see: Wang *et al.* (2012 ▶); Horkaew *et al.* (2012 ▶); Li (2011*a* ▶,*b* ▶, 2012 ▶).
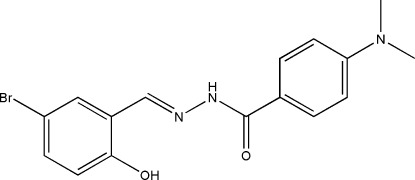


## Experimental

### 

#### Crystal data

C_16_H_16_BrN_3_O_2_
*M* *_r_* = 362.23
Monoclinic, 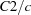

*a* = 35.3800 (12) Å
*b* = 10.452 (1) Å
*c* = 18.5070 (15) Å
β = 111.463 (2)°
*V* = 6369.1 (8) Å^3^
*Z* = 16
Mo *K*α radiation
μ = 2.59 mm^−1^
*T* = 298 K
0.20 × 0.20 × 0.18 mm

#### Data collection

Bruker SMART CCD area-detector diffractometer
Absorption correction: multi-scan (*SADABS*; Sheldrick, 1996 ▶) *T*_min_ = 0.625, *T*_max_ = 0.653
25287 measured reflections
6931 independent reflections
2320 reflections with *I* > 2σ(*I*)
*R*_int_ = 0.168

#### Refinement

*R*[*F* ^2^ > 2σ(*F* ^2^)] = 0.065
*wR*(*F* ^2^) = 0.197
*S* = 0.94
6931 reflections
411 parameters
3 restraints
H atoms treated by a mixture of independent and constrained refinement
Δρ_max_ = 0.70 e Å^−3^
Δρ_min_ = −0.44 e Å^−3^

### 

Data collection: *SMART* (Bruker, 1998 ▶); cell refinement: *SAINT* (Bruker, 1998 ▶); data reduction: *SAINT*; program(s) used to solve structure: *SHELXS97* (Sheldrick, 2008 ▶); program(s) used to refine structure: *SHELXL97* (Sheldrick, 2008 ▶); molecular graphics: *SHELXTL* (Sheldrick, 2008 ▶); software used to prepare material for publication: *SHELXTL*.

## Supplementary Material

Crystal structure: contains datablock(s) global, I. DOI: 10.1107/S1600536812013785/su2400sup1.cif

Structure factors: contains datablock(s) I. DOI: 10.1107/S1600536812013785/su2400Isup2.hkl

Supplementary material file. DOI: 10.1107/S1600536812013785/su2400Isup3.cml

Additional supplementary materials:  crystallographic information; 3D view; checkCIF report

## Figures and Tables

**Table 1:** Hydrogen-bond geometry (Å, °)

*D*—H⋯*A*	*D*—H	H⋯*A*	*D*⋯*A*	*D*—H⋯*A*
O1—H1⋯N1	0.85 (1)	1.78 (3)	2.570 (7)	153 (7)
O3—H3*A*⋯N4	0.82	1.87	2.588 (7)	146
N2—H2⋯O4	0.90 (1)	2.04 (2)	2.920 (6)	166 (6)
N5—H5⋯O2^i^	0.90 (1)	1.95 (3)	2.807 (7)	160 (6)

Symmetry code: (i) 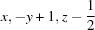.

**Fig. 1.**
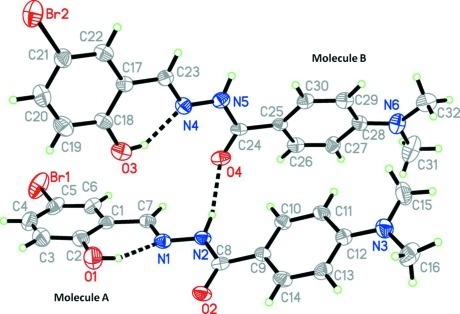
The molecular structure of the two indpendent molecules (A and B) of the title compound, showing the atom labelling. The displacement ellipsoids are drawn at the 30% probability level. Hydrogen bonds are drawn as dashed lines (see Table 1 for details).

**Fig. 2.**
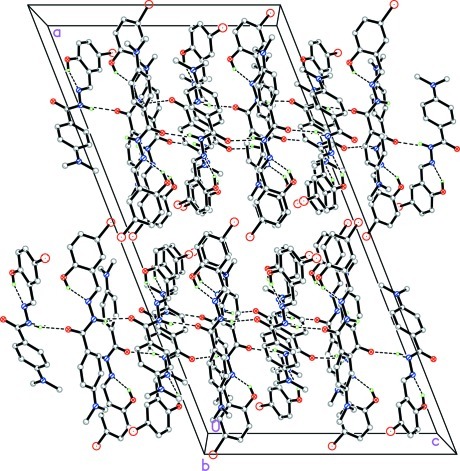
Crystal packing diagram of the title compound, viewed along the b axis. Hydrogen bonds are drawn as dashed lines (see Table 1 for details; C bound H atoms have been omitted for clarity).

## supplementary crystallographic information

### Comment

Continuing our work on the preparation (*E*)-*N*'-(5-Bromo-2-hydroxybenzylidene)-4-(dimethylamino)benzohydrazideof benzohydrazone compounds, the new title compound was synthesized and we report herein on its crystal structure.

The asymmetric unit of the title compound contains two independent molecules (A and B), Fig. 1. Each molecule has an *E* conformation about the C═N bond. In molecule A the dihedral angle between the C1–C6 and C9–C14 benzene rings is 30.5 (3)°. In molecule B the dihedral angle between the benzene rings C17–C22 and C25–C30 is 28.7 (3)°. The bond lengths are within normal values when compared with those observed in the similar compounds (Wang *et al.*, 2012; Horkaew *et al.*, 2012; Li, 2011*a*,*b*; Li, 2012). In each molecule there is an O-H···N hydrogen bond , and the two indpendent molecules are linked by an N-H···O hydrogen bond (Table 1 and Fig. 1).

In the crystal, molecules are linked through N–H···O hydrogen bonds to form chains propagating along the *c* axis direction (Table 1 and Fig. 2).

### Experimental

A mixture of 5-bromosalicylaldehyde (0.201 g, 1 mmol) and 4-dimethylaminobenzohydrazide (0.179 g, 1 mmol), and a few drops of acetic acid, were mixed and refluxed in 20 ml ethanol for 30 min. The reaction mixture was then cooled slowly to room temperature. Colourless block-like crystals of the title compound, suitable for X-ray analysis, were formed by slow evaporation of the solution.

### Refinement

The H atoms H1, H2, and H5 were located from a difference Fourier map and were freely refined. The remaining H-atoms were positioned geometrically and refined using a riding model: O–H = 0.82 Å, C–H = 0.93 and 0.96 Å, for CH and CH_3_ H atoms, respectively, with *U*_iso_(H) = k × *U*_eq_(O,C) where k = 1.5 for and OH and CH_3_ H atoms, and = 1.2 for other H atoms.

### Crystal data

**Table:**

C_16_H_16_BrN_3_O_2_	*F*(000) = 2944
*M**_r_* = 362.23	*D*_x_ = 1.511 Mg m^−^^3^
Monoclinic, *C*2/*c*	Mo *K*α radiation, λ = 0.71073 Å
Hall symbol: -C 2yc	Cell parameters from 1759 reflections
*a* = 35.3800 (12) Å	θ = 2.3–24.3°
*b* = 10.452 (1) Å	µ = 2.59 mm^−^^1^
*c* = 18.5070 (15) Å	*T* = 298 K
β = 111.463 (2)°	Block, colourless
*V* = 6369.1 (8) Å^3^	0.20 × 0.20 × 0.18 mm
*Z* = 16	

### Data collection

**Table:**

Bruker SMART CCD area-detector diffractometer	6931 independent reflections
Radiation source: fine-focus sealed tube	2320 reflections with *I* > 2σ(*I*)
Graphite monochromator	*R*_int_ = 0.168
ω scans	θ_max_ = 27.0°, θ_min_ = 2.3°
Absorption correction: multi-scan (*SADABS*; Sheldrick, 1996)	*h* = −43→45
*T*_min_ = 0.625, *T*_max_ = 0.653	*k* = −13→12
25287 measured reflections	*l* = −23→23

### Refinement

**Table:**

Refinement on *F*^2^	Primary atom site location: structure-invariant direct methods
Least-squares matrix: full	Secondary atom site location: difference Fourier map
*R*[*F*^2^ > 2σ(*F*^2^)] = 0.065	Hydrogen site location: inferred from neighbouring sites
*wR*(*F*^2^) = 0.197	H atoms treated by a mixture of independent and constrained refinement
*S* = 0.94	*w* = 1/[σ^2^(*F*_o_^2^) + (0.0705*P*)^2^] where *P* = (*F*_o_^2^ + 2*F*_c_^2^)/3
6931 reflections	(Δ/σ)_max_ < 0.001
411 parameters	Δρ_max_ = 0.70 e Å^−^^3^
3 restraints	Δρ_min_ = −0.44 e Å^−^^3^

### Special details

**Table:**

Geometry. All e.s.d.'s (except the e.s.d. in the dihedral angle between two l.s. planes) are estimated using the full covariance matrix. The cell e.s.d.'s are taken into account individually in the estimation of e.s.d.'s in distances, angles and torsion angles; correlations between e.s.d.'s in cell parameters are only used when they are defined by crystal symmetry. An approximate (isotropic) treatment of cell e.s.d.'s is used for estimating e.s.d.'s involving l.s. planes.
Refinement. Refinement of *F*^2^ against ALL reflections. The weighted *R*-factor *wR* and goodness of fit *S* are based on *F*^2^, conventional *R*-factors *R* are based on *F*, with *F* set to zero for negative *F*^2^. The threshold expression of *F*^2^ > σ(*F*^2^) is used only for calculating *R*-factors(gt) *etc*. and is not relevant to the choice of reflections for refinement. *R*-factors based on *F*^2^ are statistically about twice as large as those based on *F*, and *R*- factors based on ALL data will be even larger.

### Fractional atomic coordinates and isotropic or equivalent isotropic displacement parameters (Å^2^)

**Table:**

	*x*	*y*	*z*	*U*_iso_*/*U*_eq_	
Br1	0.05924 (3)	−0.17985 (8)	0.26798 (6)	0.1074 (4)	
O1	0.09419 (15)	0.3023 (6)	0.4577 (3)	0.0859 (16)	
O2	0.19862 (13)	0.4457 (4)	0.5806 (2)	0.0575 (12)	
N1	0.16745 (16)	0.2904 (5)	0.4615 (3)	0.0483 (14)	
N2	0.20495 (17)	0.3485 (5)	0.4771 (3)	0.0488 (14)	
N3	0.34499 (18)	0.7781 (5)	0.5532 (3)	0.0642 (16)	
C1	0.1174 (2)	0.1398 (6)	0.3926 (3)	0.0457 (16)	
C2	0.0872 (2)	0.1945 (7)	0.4124 (4)	0.0616 (19)	
C3	0.0489 (3)	0.1427 (9)	0.3884 (4)	0.081 (2)	
H3	0.0285	0.1829	0.4005	0.097*	
C4	0.0410 (2)	0.0325 (9)	0.3468 (4)	0.078 (2)	
H4	0.0153	−0.0046	0.3319	0.094*	
C5	0.0707 (3)	−0.0244 (7)	0.3268 (4)	0.067 (2)	
C6	0.1089 (2)	0.0274 (6)	0.3481 (4)	0.0601 (19)	
H6	0.1286	−0.0112	0.3333	0.072*	
C7	0.15762 (19)	0.1970 (6)	0.4126 (4)	0.0487 (17)	
H7	0.1757	0.1668	0.3907	0.058*	
C8	0.2167 (2)	0.4381 (6)	0.5350 (4)	0.0462 (16)	
C9	0.25160 (19)	0.5209 (6)	0.5393 (3)	0.0421 (15)	
C10	0.27268 (19)	0.5113 (6)	0.4902 (3)	0.0473 (16)	
H10	0.2659	0.4464	0.4533	0.057*	
C11	0.30347 (19)	0.5941 (6)	0.4937 (3)	0.0513 (17)	
H11	0.3172	0.5836	0.4599	0.062*	
C12	0.31442 (19)	0.6953 (6)	0.5485 (4)	0.0468 (16)	
C13	0.2933 (2)	0.7025 (6)	0.5984 (4)	0.0570 (19)	
H13	0.3003	0.7656	0.6365	0.068*	
C14	0.2622 (2)	0.6194 (6)	0.5936 (4)	0.0540 (18)	
H14	0.2482	0.6294	0.6270	0.065*	
C15	0.3659 (2)	0.7698 (7)	0.5001 (5)	0.088 (3)	
H15A	0.3793	0.6885	0.5060	0.132*	
H15B	0.3856	0.8372	0.5108	0.132*	
H15C	0.3467	0.7781	0.4479	0.132*	
C16	0.3560 (2)	0.8819 (6)	0.6096 (4)	0.082 (2)	
H16A	0.3324	0.9323	0.6037	0.123*	
H16B	0.3762	0.9348	0.6012	0.123*	
H16C	0.3668	0.8470	0.6612	0.123*	
Br2	−0.01643 (2)	0.70242 (8)	0.01969 (5)	0.0909 (4)	
O3	0.10533 (15)	0.3944 (4)	0.2737 (3)	0.0643 (13)	
H3A	0.1282	0.4005	0.2723	0.097*	
O4	0.21854 (13)	0.3436 (4)	0.3307 (2)	0.0555 (12)	
N4	0.15959 (17)	0.4691 (5)	0.2196 (3)	0.0499 (14)	
N5	0.19915 (17)	0.4732 (5)	0.2255 (3)	0.0520 (14)	
N6	0.39439 (18)	0.5341 (5)	0.3585 (3)	0.0650 (16)	
C17	0.0911 (2)	0.5253 (6)	0.1589 (4)	0.0453 (16)	
C18	0.0787 (2)	0.4598 (6)	0.2135 (4)	0.0508 (17)	
C19	0.0387 (3)	0.4636 (7)	0.2065 (5)	0.072 (2)	
H19	0.0306	0.4175	0.2414	0.086*	
C20	0.0101 (2)	0.5342 (8)	0.1489 (5)	0.075 (2)	
H20	−0.0168	0.5370	0.1456	0.090*	
C21	0.0222 (2)	0.6009 (7)	0.0961 (4)	0.0591 (19)	
C22	0.0617 (2)	0.5949 (6)	0.1007 (4)	0.0533 (18)	
H22	0.0691	0.6384	0.0641	0.064*	
C23	0.1324 (2)	0.5246 (6)	0.1638 (4)	0.0492 (17)	
H23	0.1394	0.5653	0.1257	0.059*	
C24	0.2283 (2)	0.4141 (6)	0.2863 (4)	0.0445 (16)	
C25	0.27063 (18)	0.4424 (5)	0.2981 (3)	0.0389 (15)	
C26	0.3015 (2)	0.3623 (6)	0.3429 (3)	0.0506 (17)	
H26	0.2944	0.2859	0.3604	0.061*	
C27	0.3415 (2)	0.3897 (6)	0.3625 (4)	0.0552 (18)	
H27	0.3608	0.3324	0.3933	0.066*	
C28	0.3544 (2)	0.5040 (6)	0.3371 (4)	0.0466 (16)	
C29	0.3231 (2)	0.5837 (6)	0.2899 (4)	0.0532 (18)	
H29	0.3299	0.6590	0.2707	0.064*	
C30	0.2829 (2)	0.5538 (6)	0.2713 (3)	0.0494 (17)	
H30	0.2633	0.6094	0.2398	0.059*	
C31	0.4259 (2)	0.4599 (8)	0.4144 (5)	0.104 (3)	
H31A	0.4193	0.4464	0.4597	0.155*	
H31B	0.4513	0.5050	0.4288	0.155*	
H31C	0.4283	0.3788	0.3921	0.155*	
C32	0.4071 (2)	0.6560 (6)	0.3366 (4)	0.069 (2)	
H32A	0.3908	0.6751	0.2836	0.103*	
H32B	0.4351	0.6508	0.3420	0.103*	
H32C	0.4039	0.7224	0.3697	0.103*	
H2	0.2137 (19)	0.345 (6)	0.437 (3)	0.080*	
H5	0.2045 (19)	0.509 (6)	0.186 (3)	0.080*	
H1	0.1197 (5)	0.314 (7)	0.471 (4)	0.080*	

### Atomic displacement parameters (Å^2^)

**Table:**

	*U*^11^	*U*^22^	*U*^33^	*U*^12^	*U*^13^	*U*^23^
Br1	0.0913 (7)	0.0587 (6)	0.1314 (9)	−0.0037 (5)	−0.0073 (6)	−0.0273 (5)
O1	0.075 (4)	0.097 (4)	0.096 (4)	−0.017 (4)	0.043 (4)	−0.043 (4)
O2	0.077 (3)	0.058 (3)	0.047 (3)	−0.006 (2)	0.034 (3)	−0.007 (2)
N1	0.061 (4)	0.041 (3)	0.044 (3)	−0.002 (3)	0.021 (3)	0.002 (3)
N2	0.062 (4)	0.049 (3)	0.040 (3)	−0.005 (3)	0.024 (3)	−0.005 (3)
N3	0.069 (4)	0.059 (4)	0.068 (4)	−0.013 (3)	0.029 (4)	−0.004 (3)
C1	0.053 (5)	0.045 (4)	0.037 (4)	0.000 (4)	0.014 (3)	0.007 (3)
C2	0.066 (5)	0.065 (5)	0.056 (5)	−0.008 (5)	0.025 (4)	−0.008 (4)
C3	0.070 (6)	0.103 (7)	0.074 (6)	−0.010 (5)	0.033 (5)	−0.011 (5)
C4	0.061 (5)	0.090 (7)	0.073 (6)	−0.016 (5)	0.011 (5)	0.004 (5)
C5	0.077 (6)	0.047 (5)	0.058 (5)	−0.011 (4)	0.001 (4)	−0.004 (4)
C6	0.067 (5)	0.052 (5)	0.051 (4)	0.001 (4)	0.010 (4)	−0.001 (4)
C7	0.056 (5)	0.047 (4)	0.043 (4)	0.011 (4)	0.019 (3)	0.008 (4)
C8	0.060 (4)	0.044 (4)	0.033 (4)	0.009 (4)	0.015 (3)	0.001 (3)
C9	0.052 (4)	0.037 (4)	0.040 (4)	0.005 (3)	0.020 (3)	0.000 (3)
C10	0.059 (4)	0.042 (4)	0.046 (4)	−0.001 (4)	0.025 (4)	−0.007 (3)
C11	0.061 (5)	0.057 (5)	0.045 (4)	−0.003 (4)	0.031 (4)	−0.011 (4)
C12	0.054 (4)	0.037 (4)	0.049 (4)	0.001 (4)	0.017 (4)	−0.001 (3)
C13	0.075 (5)	0.043 (4)	0.048 (4)	−0.009 (4)	0.017 (4)	−0.015 (3)
C14	0.075 (5)	0.045 (4)	0.051 (4)	−0.001 (4)	0.033 (4)	−0.004 (4)
C15	0.079 (6)	0.092 (6)	0.102 (6)	−0.037 (5)	0.044 (5)	−0.015 (5)
C16	0.102 (6)	0.043 (4)	0.082 (6)	−0.016 (4)	0.012 (5)	−0.010 (4)
Br2	0.0675 (6)	0.0902 (7)	0.1043 (7)	0.0140 (5)	0.0188 (5)	0.0122 (5)
O3	0.083 (4)	0.052 (3)	0.064 (3)	−0.001 (3)	0.034 (3)	0.003 (3)
O4	0.072 (3)	0.050 (3)	0.044 (3)	−0.016 (2)	0.021 (2)	0.000 (2)
N4	0.050 (4)	0.047 (3)	0.056 (4)	−0.005 (3)	0.023 (3)	−0.003 (3)
N5	0.060 (4)	0.052 (4)	0.045 (4)	−0.003 (3)	0.020 (3)	0.012 (3)
N6	0.058 (4)	0.054 (4)	0.080 (4)	−0.002 (3)	0.022 (3)	0.016 (3)
C17	0.053 (4)	0.034 (4)	0.048 (4)	−0.005 (3)	0.018 (4)	−0.004 (3)
C18	0.064 (5)	0.039 (4)	0.058 (5)	−0.009 (4)	0.032 (4)	−0.010 (4)
C19	0.080 (6)	0.071 (5)	0.080 (6)	0.000 (5)	0.046 (5)	0.007 (5)
C20	0.060 (5)	0.084 (6)	0.091 (6)	0.001 (5)	0.039 (5)	−0.001 (5)
C21	0.052 (5)	0.059 (5)	0.065 (5)	−0.004 (4)	0.021 (4)	−0.007 (4)
C22	0.065 (5)	0.041 (4)	0.060 (5)	−0.011 (4)	0.030 (4)	−0.008 (4)
C23	0.051 (4)	0.050 (4)	0.045 (4)	0.000 (4)	0.015 (4)	−0.001 (3)
C24	0.062 (5)	0.039 (4)	0.033 (4)	−0.004 (4)	0.018 (4)	−0.005 (3)
C25	0.052 (4)	0.036 (4)	0.032 (4)	0.000 (3)	0.019 (3)	0.006 (3)
C26	0.075 (5)	0.026 (4)	0.051 (4)	−0.002 (4)	0.023 (4)	0.001 (3)
C27	0.057 (5)	0.043 (4)	0.060 (5)	0.007 (4)	0.016 (4)	0.005 (4)
C28	0.059 (5)	0.033 (4)	0.050 (4)	−0.001 (4)	0.022 (4)	0.000 (3)
C29	0.073 (5)	0.036 (4)	0.053 (4)	−0.002 (4)	0.025 (4)	0.007 (3)
C30	0.064 (5)	0.039 (4)	0.043 (4)	0.002 (3)	0.017 (3)	0.012 (3)
C31	0.069 (6)	0.081 (6)	0.135 (8)	−0.005 (5)	0.007 (5)	0.029 (6)
C32	0.080 (5)	0.059 (5)	0.068 (5)	−0.017 (4)	0.029 (4)	0.007 (4)

### Geometric parameters (Å, º)

**Table:**

Br1—C5	1.915 (7)	Br2—C21	1.893 (7)
O1—C2	1.372 (8)	O3—C18	1.352 (7)
O1—H1	0.852 (10)	O3—H3A	0.8200
O2—C8	1.232 (7)	O4—C24	1.244 (7)
N1—C7	1.290 (7)	N4—C23	1.266 (7)
N1—N2	1.390 (7)	N4—N5	1.364 (7)
N2—C8	1.369 (7)	N5—C24	1.364 (7)
N2—H2	0.900 (10)	N5—H5	0.900 (10)
N3—C12	1.363 (7)	N6—C28	1.358 (7)
N3—C15	1.432 (8)	N6—C31	1.440 (8)
N3—C16	1.456 (8)	N6—C32	1.457 (8)
C1—C2	1.375 (9)	C17—C22	1.396 (8)
C1—C6	1.403 (8)	C17—C18	1.417 (8)
C1—C7	1.460 (8)	C17—C23	1.432 (8)
C2—C3	1.375 (9)	C18—C19	1.373 (9)
C3—C4	1.356 (10)	C19—C20	1.385 (9)
C3—H3	0.9300	C19—H19	0.9300
C4—C5	1.370 (10)	C20—C21	1.391 (9)
C4—H4	0.9300	C20—H20	0.9300
C5—C6	1.374 (9)	C21—C22	1.368 (8)
C6—H6	0.9300	C22—H22	0.9300
C7—H7	0.9300	C23—H23	0.9300
C8—C9	1.487 (8)	C24—C25	1.462 (8)
C9—C10	1.373 (8)	C25—C26	1.384 (8)
C9—C14	1.391 (8)	C25—C30	1.396 (8)
C10—C11	1.374 (8)	C26—C27	1.356 (8)
C10—H10	0.9300	C26—H26	0.9300
C11—C12	1.418 (8)	C27—C28	1.420 (8)
C11—H11	0.9300	C27—H27	0.9300
C12—C13	1.387 (9)	C28—C29	1.406 (8)
C13—C14	1.377 (8)	C29—C30	1.371 (8)
C13—H13	0.9300	C29—H29	0.9300
C14—H14	0.9300	C30—H30	0.9300
C15—H15A	0.9600	C31—H31A	0.9600
C15—H15B	0.9600	C31—H31B	0.9600
C15—H15C	0.9600	C31—H31C	0.9600
C16—H16A	0.9600	C32—H32A	0.9600
C16—H16B	0.9600	C32—H32B	0.9600
C16—H16C	0.9600	C32—H32C	0.9600
			
C2—O1—H1	104 (5)	C18—O3—H3A	109.5
C7—N1—N2	117.9 (5)	C23—N4—N5	120.0 (5)
C8—N2—N1	116.9 (5)	N4—N5—C24	119.7 (5)
C8—N2—H2	125 (5)	N4—N5—H5	118 (4)
N1—N2—H2	114 (4)	C24—N5—H5	122 (4)
C12—N3—C15	121.0 (6)	C28—N6—C31	122.3 (6)
C12—N3—C16	121.1 (6)	C28—N6—C32	121.1 (6)
C15—N3—C16	117.8 (6)	C31—N6—C32	115.7 (6)
C2—C1—C6	118.9 (6)	C22—C17—C18	117.7 (6)
C2—C1—C7	122.8 (6)	C22—C17—C23	120.4 (6)
C6—C1—C7	118.3 (6)	C18—C17—C23	121.9 (6)
O1—C2—C1	121.5 (7)	O3—C18—C19	118.4 (7)
O1—C2—C3	117.1 (7)	O3—C18—C17	121.8 (6)
C1—C2—C3	121.4 (7)	C19—C18—C17	119.7 (7)
C4—C3—C2	119.5 (8)	C18—C19—C20	121.7 (7)
C4—C3—H3	120.2	C18—C19—H19	119.1
C2—C3—H3	120.2	C20—C19—H19	119.1
C3—C4—C5	120.1 (7)	C19—C20—C21	118.7 (7)
C3—C4—H4	119.9	C19—C20—H20	120.6
C5—C4—H4	119.9	C21—C20—H20	120.6
C4—C5—C6	121.6 (7)	C22—C21—C20	120.3 (7)
C4—C5—Br1	119.9 (6)	C22—C21—Br2	120.9 (6)
C6—C5—Br1	118.5 (7)	C20—C21—Br2	118.7 (6)
C5—C6—C1	118.4 (7)	C21—C22—C17	121.8 (6)
C5—C6—H6	120.8	C21—C22—H22	119.1
C1—C6—H6	120.8	C17—C22—H22	119.1
N1—C7—C1	118.4 (6)	N4—C23—C17	120.4 (6)
N1—C7—H7	120.8	N4—C23—H23	119.8
C1—C7—H7	120.8	C17—C23—H23	119.8
O2—C8—N2	119.7 (6)	O4—C24—N5	120.2 (6)
O2—C8—C9	123.7 (6)	O4—C24—C25	122.6 (6)
N2—C8—C9	116.6 (6)	N5—C24—C25	117.1 (6)
C10—C9—C14	117.6 (6)	C26—C25—C30	115.9 (6)
C10—C9—C8	124.3 (6)	C26—C25—C24	120.5 (6)
C14—C9—C8	118.0 (6)	C30—C25—C24	123.4 (6)
C9—C10—C11	122.4 (6)	C27—C26—C25	123.4 (6)
C9—C10—H10	118.8	C27—C26—H26	118.3
C11—C10—H10	118.8	C25—C26—H26	118.3
C10—C11—C12	120.5 (6)	C26—C27—C28	121.4 (6)
C10—C11—H11	119.7	C26—C27—H27	119.3
C12—C11—H11	119.7	C28—C27—H27	119.3
N3—C12—C13	122.2 (6)	N6—C28—C29	122.8 (6)
N3—C12—C11	121.5 (6)	N6—C28—C27	121.8 (6)
C13—C12—C11	116.3 (6)	C29—C28—C27	115.3 (6)
C14—C13—C12	122.3 (6)	C30—C29—C28	122.0 (6)
C14—C13—H13	118.9	C30—C29—H29	119.0
C12—C13—H13	118.9	C28—C29—H29	119.0
C13—C14—C9	120.8 (6)	C29—C30—C25	122.1 (6)
C13—C14—H14	119.6	C29—C30—H30	119.0
C9—C14—H14	119.6	C25—C30—H30	119.0
N3—C15—H15A	109.5	N6—C31—H31A	109.5
N3—C15—H15B	109.5	N6—C31—H31B	109.5
H15A—C15—H15B	109.5	H31A—C31—H31B	109.5
N3—C15—H15C	109.5	N6—C31—H31C	109.5
H15A—C15—H15C	109.5	H31A—C31—H31C	109.5
H15B—C15—H15C	109.5	H31B—C31—H31C	109.5
N3—C16—H16A	109.5	N6—C32—H32A	109.5
N3—C16—H16B	109.5	N6—C32—H32B	109.5
H16A—C16—H16B	109.5	H32A—C32—H32B	109.5
N3—C16—H16C	109.5	N6—C32—H32C	109.5
H16A—C16—H16C	109.5	H32A—C32—H32C	109.5
H16B—C16—H16C	109.5	H32B—C32—H32C	109.5

### Hydrogen-bond geometry (Å, º)

**Table:**

*D*—H···*A*	*D*—H	H···*A*	*D*···*A*	*D*—H···*A*
O1—H1···N1	0.85 (1)	1.78 (3)	2.570 (7)	153 (7)
O3—H3*A*···N4	0.82	1.87	2.588 (7)	146
N2—H2···O4	0.90 (1)	2.04 (2)	2.920 (6)	166 (6)
N5—H5···O2^i^	0.90 (1)	1.95 (3)	2.807 (7)	160 (6)

Symmetry code: (i) *x*, −*y*+1, *z*−1/2.
